# Clinical Study on Corneal Topographical Changes in Vernal Keratoconjunctivitis by Using OCULUS Pentacam®

**DOI:** 10.7759/cureus.33798

**Published:** 2023-01-15

**Authors:** Dharshana Thiagarajan, Safira Zainal, Rohanah Alias, Mae-Lynn Catherine Bastion

**Affiliations:** 1 Ophthalmology, Universiti Kebangsaan Malaysia Medical Centre, Kuala Lumpur, MYS; 2 Ophthalmology, Hospital Kuala Lumpur, Kuala Lumpur, MYS; 3 Ophthalmology, Hospital Universiti Kebangsaan Malaysia, Kuala Lumpur, MYS

**Keywords:** vkc, keratoconus, vernal keratoconjunctivitis, pentacam, corneal topography

## Abstract

Purpose: To evaluate the corneal topographical changes in vernal keratoconjunctivitis (VKC) subjects using OCULUS Pentacam.

Design: This was a cross-sectional study.

Methods: VKC patients and normal subjects who fulfilled the inclusion and exclusion criteria were recruited by convenience sampling into the study. Subjects underwent a best-corrected visual acuity measurement with a Snellen chart, retinoscopy, and corneal topography (OCULUS Pentacam®), followed by anterior segment and fundus examination and intraocular pressure measurement. Data were collected and analyzed using SPSS version 26.0 for Windows (SPSS Inc. Chicago, IL, USA). A p-value <0.05 was considered statistically significant.

Results: A total of 78 eyes of 43 VKC patients and 84 eyes of normal subjects were included in the study. Most of the VKC subjects were Malay males aged 10 years or less. A majority (71.8%) had palpebral VKC of five years duration or less (57.7%) and presented between the ages of six and 10 years (44.9%). Central corneal curvature and astigmatism were significantly higher in VKC subjects compared to the normal population (p < 0.05). The minimal pachymetry was significantly lower with a longer duration of VKC (p < 0.05). Older age of presentation of VKC was associated with higher central corneal curvatures and thinner minimal pachymetry (p < 0.05). There was no association between the type of VKC and corneal topography changes. The prevalence of keratoconus and subclinical keratoconus among VKC subjects was 10.3% and 11.5%, respectively.

Conclusion: Longer duration and older age of presentation of VKC are associated with significant corneal topographical changes, thus exposing them to a higher risk of the future development of keratoconus.

## Introduction

Vernal keratoconjunctivitis (VKC) is a subtype of allergic conjunctival disease affecting the ocular surface and is characterized by chronic, bilateral inflammation of the upper tarsal and or limbal conjunctiva [1]. VKC is seen in young males commonly living in hot, dry climates such as West Africa, the Mediterranean basin, the Middle East, Japan, India, and South America [2-5]. Despite usually resolving after puberty, severe forms of the disease may cause permanent visual impairment [1].

Corneal involvement in VKC involves injury to the superficial and basal epithelial layers and anterior stroma [1]. Corneal nerves in these patients are less sensitive and have a higher concentration of surrounding inflammatory cells [6]. Excessive eye rubbing and a slow release of matrix metalloproteinases penetrate the cornea in the presence of chronic epithelial damage causing high astigmatism and corneal ectasia [7,8]. These corneal changes may be subtle in the early stages and progress undetected until advanced complications such as keratoconus develop. Keratoconus is the most common ectatic disorder in VKC, causing progressive thinning and protrusion of the cornea leading to progressive myopia and irregular astigmatism [9].

Early detection of these subtle corneal changes is vital to help arrest these changes in the early stages before developing further complications. These changes can be measured accurately by corneal topography, making it the gold standard in the screening of keratoconus and other corneal ectasias [10]. OCULUS Pentacam® is a rotating Scheimpflug camera tomographer that enables quick anterior segment image generation in three dimensions. It allows measurement of the elevation and curvature of both the anterior and posterior corneal surfaces and pachymetry compared to traditional Placido-based topographers, which only measure the curvature of the anterior corneal surface [10]. This is important as the back difference elevation (BDE) has recently been determined as an excellent predictor of keratoconus when used in conjunction with other corneal parameters [11]. Elevation-based corneal topographers also measure a larger area of cornea (8-9 mm in diameter) compared to Placido-based topographers (6 mm) allowing the detection of more peripheral corneal changes [12].

Most of the studies on VKC subjects were done using Placido-based corneal topographers. These studies showed the incidence of keratoconus-like topography (KLT), keratoconus, and subclinical keratoconus by corneal topography to be significantly higher than those diagnosed clinically [13-16]. The central corneal thickness was found to be reduced in VKC and even more reduced in those with KLT [13]. VKC subjects were also found to have higher corneal curvatures and astigmatism than normal subjects [13,15]. The incidence of keratoconus in VKC patients was associated with male gender, long-standing disease, mixed and palpebral forms, and advanced corneal lesions [14]. One study found more significant corneal curvature changes associated with a longer duration of symptoms [16]. However, Lapid-Gortzak et al. found no correlation between the type, severity, and duration of VKC with corneal topographical changes [15].

In a study using slit-scanning topography by Baretto et al., eyes with VKC had higher elevation peaks both anteriorly and posteriorly with thinner corneas, increased central corneal curvatures, and more abnormal pachymetric indices compared to normal eyes. Twenty percent of VKC subjects had keratoconus, whereas a further 14% were found to have subclinical keratoconus [17].

To our knowledge, no studies on VKC were conducted using Scheimpflug photography-based corneal topographers, and there are limited data on elevation-based corneal changes in VKC subjects. There are also scarce data on the corneal topographical changes in VKC based on type, duration, and age of presentation, which is valuable in clinical practice. There are no studies to determine the prevalence of keratoconus and subclinical keratoconus among VKC patients in Malaysia. Hence, we embarked on this study to determine the corneal topographical changes and prevalence of keratoconus and subclinical keratoconus in VKC among our local population by using OCULUS Pentacam®, identify those who are at a higher risk for the development of keratoconus, and establish the necessity for early corneal topography in these patients. Early detection of corneal topographical changes may allow more aggressive treatment options to prevent progression and significant ocular morbidity in these patients.

## Materials and methods

This was a cross-sectional study conducted between October 2021 and January 2022 at the ophthalmology clinic of Hospital Kuala Lumpur (HKL). VKC patients on follow-up and normal subjects who presented to the ophthalmology clinic of HKL who fulfilled the inclusion criteria were recruited. The study commenced after obtaining research approval from the Medical Research and Ethical Committees of both Universiti Kebangsaan Malaysia (UKM PPI/111/8/JEP-2020-816) and the Ministry of Health (KKM/NIHSEC/P20-2311(11), KKM/NIHSEC/P20-2311) and conducted following the Declaration of Helsinki.

Convenient sampling was performed for VKC patients and normal subjects (age and gender matched). The diagnosis of VKC had already been established by the consultant corneal ophthalmologist. The allergens were established with either immunoglobulin E immunoassay or skin testing. Types of VKC were divided into palpebral, limbal, and mixed based on the clinical classification by Emmert. Normal subjects were those with no ocular or systemic comorbidities, no allergies, no atopy, and with a best-corrected visual acuity (BCVA) of 6/6 and a refractive error ≤-3.00D sphere and ≤-1.75D cylinder. The inclusion criteria included all VKC patients and a comparative group of normal subjects who presented to the ophthalmology clinic of HKL during the study period. Exclusion criteria included patients unable to maintain the position for corneal topography and slit lamp examination, patients with contact lens usage within two weeks of examination, active ocular infection, and past history of ocular surgery or trauma, pregnant or lactating patients as well as patients with poor ocular surface resulting in an inability to obtain an "OK" signal via OCULUS Pentacam®. VKC subjects with acute allergic conjunctivitis, previous history of shield ulcer or corneal scars, additional ocular pathology other than VKC and keratoconus, and systemic illnesses other than those related to atopy were also excluded.

A sample size of 73 eyes per group was calculated using two population means formulae based on the mean central corneal curvature (diopters) being 46.1 and 43.2 in the VKC and normal groups, respectively, with an assumption of the power of the study to be 80% at the α level of 5% [18]. Both VKC and normal subjects were age and gender matched. Eligible patients were given the patient information sheet and explained the nature of the study. Once informed written consent and assent were obtained, each subject underwent a complete ophthalmic examination. Minors below the age of 18 years were examined and had procedures performed with parents present.

During the study visit, the subject’s complete medical and ocular history was obtained, followed by a complete ophthalmic examination which included BCVA with a Snellen chart (Hamblin, London, UK), retinoscopy (Keeler), corneal topography (OCULUS Pentacam® Wetzlar, Germany), anterior and posterior segment examination with slit lamp biomicroscopy (SL-D4, Topcon, Japan) which was performed together with a consultant corneal ophthalmologist and intraocular pressure measurement using Goldman applanation tonometer mounted on the slit lamp biomicroscope under topical anesthesia. Two corneal topography measurements were taken by the same two investigators, and the results were averaged. Patients with poor ocular surfaces were treated with artificial tears for two weeks before corneal topography. Only patients with scans obtaining acceptable "OK" signals on OCULUS Pentacam® were included.

The parameters that were measured were the flattest meridian in the central 3 mm zone (K1), the steepest meridian in the central 3 mm zone (K2), mean corneal power in the 3 mm zone (Km), astigmatism, corneal thickness at the thinnest point of the cornea (minimal pachymetry), front difference elevation (FDE), and BDE. The best-fit sphere (BFS) is the central 8 mm optical zone of the cornea, which is the commonly used reference surface to measure elevation data. An "enhanced BFS" subtracts a 4 mm zone centered on the thinnest point of the cornea from the BFS, thus maximizing the elevation difference between the apex of the cone and the rest of the peripheral cornea. The anterior and posterior elevations were thus measured by subtracting the difference in elevation between the "enhanced BFS" and the BFS.

Keratoconus was defined as an eye with a characteristic slit lamp and keratometric or retinoscopic findings of keratoconus with characteristic corneal topographic abnormalities: FDE ≥ 12 μm or BDE ≥ 20 μm with corneal thickness <500 μm and Km >47 D [19]. An eye was said to have subclinical keratoconus when the corneal topography showed FDE between 6 and 12 μm or BDE between 8 and 20 μm with a corresponding corneal thickness of <500 μm but without characteristic slit lamp, keratometric, or retinoscopic findings of keratoconus [19].

Statistical analysis was performed using Statistical Package for Social Science (SPSS), version 26.0 for Windows (SPSS Inc. Chicago, IL, USA). Seventy-eight eyes of 43 VKC patients and 84 eyes of 43 normal subjects were included in the final analysis.

All variables were defined by descriptive analysis. The analysis of quantitative variables included a calculation of the median and interquartile range (IQR) as the data were not normally distributed. Categorical data, which were gender, age, ethnicity, laterality, and incidence of keratoconus and subclinical keratoconus, were expressed as frequencies (n) and percentages (%). The corneal topographical changes between VKC and normal subjects were analyzed using the Mann-Whitney test. The association between corneal topographical changes and type, duration, and age of presentation of VKC were analyzed using the Kruskal-Wallis test. A p-value of <0.05 was considered statistically significant.

## Results

There was a total of 78 eyes of 43 VKC patients and 84 eyes of 43 normal subjects recruited in the study with no dropout. The median age of VKC and normal subjects was 14 and 15 years, respectively. Most subjects in both groups were aged 10 years or less. The age range of the subjects was 7-39 and 6-39 years for VKC and normal groups, respectively. Males outnumbered females in both groups. A majority of the subjects in both groups were Malay, followed by Chinese, Indian, and "others" which comprised one Kadazan and one Dusun subject each. There was equal distribution between the right and left eyes in both groups (Table 1).

**Table 1:** Sociodemographic characteristics of VKC and normal subjects (N = 162) VKC, vernal keratoconjunctivitis.

Variable		Result, n (%)	
		VKC (n = 78)	Normal (n = 84)
Gender	Male	53 (67.9)	50 (59.5)
	Female	25 (32.1)	34 (40.5)
Age (years)	≤10	26 (33.3)	29 (34.5)
	11-15	21 (26.9)	19 (22.6)
	16-20	17 (21.8)	16 (19.0)
	>20	14 (17.9)	20 (23.8)
Ethnicity	Malay	65 (83.3)	74 (88.1)
	Chinese	6 (7.7)	4 (4.8)
	Indian	5 (6.4)	4 (4.8)
	Others	2 (2.6)	2 (2.4)
Laterality	Right eye	41 (52.6)	42 (50.0)
	Left eye	37 (47.4)	42 (50.0)

Most subjects had palpebral VKC (71.8%), followed by mixed type (20.5%). The limbal type of VKC was the least common (7.7%). The duration of VKC was indirectly proportional to the number of patients. Most of the VKC subjects had VKC for five years or less (57.7%) and first presented between the ages of six and 10 years (44.9%). Only 6.4% of patients had VKC for more than 15 years (Table 2).

**Table 2:** Clinical characteristics of VKC subjects (N = 78) VKC, vernal keratoconjunctivitis.

Variable		Result, n (%)
Type of VKC	Palpebral	56 (71.8)
	Limbal	6 (7.7)
	Mixed	16 (20.5)
Duration of VKC (years)	0-5	45 (57.7)
	6-10	19 (24.4)
	11-15	9 (11.5)
	>15	5 (6.4)
Age of presentation (years)	0-5	10 (12.8)
	6-10	35 (44.9)
	11-15	15 (19.2)
	16-20	9 (11.5)
	21-25	9 (11.5)

The corneal topography indices between VKC and normal subjects showed significant differences in steep K2 (p = 0.001), Km (p = 0.021), and astigmatism (p < 0.001). VKC subjects had a higher steep K2, Km, and astigmatism than normal subjects (Table 3).

**Table 3:** Corneal topography indices in VKC and normal subjects (N = 162) ^a^Mann-Whitney U test. *Statistically significant. VKC, vernal keratoconjunctivitis.

Variable (median/IQR)	VKC (n = 78)	Normal (n = 84)	p-Value
Flat K_1 _(D)	42.7 (2)	42.7 (2)	0.467^a^
Steep K_2 _(D)	44.6 (3)	43.7 (2)	0.001^a^*
K_m _(D)	43.7 (2)	43.2 (2)	0.021^a^*
Minimal pachymetry (μm)	543.5 (40)	554.5 (32)	0.152^a^
Front difference elevation (μm)	+4 (4)	+4 (2)	0.457^a^
Back difference elevation (μm)	+4 (7)	+4 (4)	0.525^a^
Astigmatism (D)	-1.6 (2)	-1.1 (1)	<0.001^a^*

The limbal type of VKC consistently showed the steepest K1, K2, and Km; the highest FDE and BDE; and the thinnest minimal pachymetry. In contrast, the mixed type of VKC showed the highest corneal astigmatism. However, none of these results were statistically significant (Table 4).

**Table 4:** Corneal topography indices based on the type of VKC (N = 78) ^a^Kruskal Wallis test. VKC, vernal keratoconjunctivitis.

Variable, median (IQR)	Palpebral (n = 56)	Limbal (n = 6)	Mixed (n = 16)	p-Value
Flat K_1 _(D)	42.7 (2)	43.9 (5)	42.8 (2)	0.515^a^
Steep K_2 _(D)	44.6 (4)	45.0 (4)	44.5 (1)	0.837^a^
K_m _(D)	43.8 (2)	44.4 (4)	43.7 (1)	0.725^a^
Minimal pachymetry (μm)	543.5 (49)	542.5 (51)	569.0 (56)	0.078^a^
Front difference elevation (μm)	+4 (5)	+8.5 (14)	+3.5 (4)	0.060^a^
Back difference elevation (μm)	+4 (6)	+12 (30)	+2 (5)	0.053^a^
Astigmatism (D)	-1.6 (2)	-1.4 (1)	-2.0 (2)	0.707^a^

Longer duration of VKC was associated with a thinner minimal pachymetry (p = 0.039). Although subjects with more than 15 years of VKC showed the highest K1, K2, FDE, and astigmatism, and subjects with 6-10 years of VKC showed the highest Km and BDE, these results were not statistically significant (Table 5).

**Table 5:** Corneal topography indices based on the duration of VKC (N = 78) ^a^Kruskal-Wallis test. *Statistically significant. VKC, vernal keratoconjunctivitis.

Variable, median (IQR)	0-5 years (n = 45)	6-10 years (n = 19)	11-15 years (n = 9)	>15 years (n = 5)	p-Value
Flat K_1 _(D)	42.7 (1)	42.6 (2)	42.0 (7)	43.2 (3)	0.727^a^
Steep K_2 _(D)	44.4 (2)	45.1 (3)	43.2 (7)	45.3 (6)	0.654^a^
K_m _(D)	43.7 (2)	44.3 (3)	42.6 (7)	44.2 (5)	0.696^a^
Minimal pachymetry (μm)	546 (45)	546 (38)	539 (68)	501 (68)	0.039^a^*
Front difference elevation (μm)	+4 (4)	+5 (4)	+2 (17)	+6 (16)	0.279^a^
Back difference elevation (μm)	+3 (4)	+7 (9)	+6 (44)	+5 (37)	0.062^a^
Astigmatism (D)	-1.6 (1)	-2.2 (2)	-1.1 (2)	-2.3 (3)	0.679^a^

VKC subjects who presented late between the ages of 21 and 25 years had significantly steeper K1 (p = 0.040), K2 (p = 0.028), and Km (p = 0.014) and thinner minimal pachymetry (p = 0.006) than those who presented younger (Table 6).

**Table 6:** Corneal topography indices based on the age of presentation of VKC (N = 78) ^a^Kruskal-Wallis test. *Statistically significant. VKC, vernal keratoconjunctivitis.

Variable, median (IQR)	0-5 years (n = 10)	6-10 years (n = 35)	11-15 years (n = 15)	16-20 years (n = 9)	21-25 years (n = 9)	p-Value
Flat K_1 _(D)	42.4 (2)	42.5 (1)	43.1 (1)	43.3 (4)	45.8 (6)	0.040^a^*
Steep K_2 _(D)	44.5 (3)	44.2 (2)	45.7 (3)	45.7 (4)	46.8 (10)	0.028^a^*
K_m _(D)	43.5 (2)	43.2 (1)	44.4 (2)	44.5 (4)	46.2 (7)	0.014^a^*
Minimal pachymetry (μm)	532 (61)	546 (34)	574 (22)	542 (77)	469 (106)	0.006^a^*
Front difference elevation (μm)	+5 (4)	+4 (4)	+4 (5)	+6 (16)	+14 (24)	0.256^a^
Back difference elevation (μm)	+2.5 (6)	+4 (6)	+3 (8)	+3 (36)	+32 (43)	0.711^a^
Astigmatism (D)	-2.1 (2)	-1.5 (1)	-2.3 (2)	-1.3 (1)	-2.9 (4)	0.222^a^

The prevalence of keratoconus was highest among the palpebral type (9.0%), followed by the limbal type (1.3%). There were no cases of keratoconus among the mixed type of VKC. Similarly, the prevalence of subclinical keratoconus was highest among the palpebral type (6.4%). However, there was a higher proportion of subclinical keratoconus among the mixed type (3.8%) compared to the limbal type (1.3%) (Table 7).

**Table 7:** Prevalence of keratoconus and subclinical keratoconus among VKC subjects (N = 78) VKC, vernal keratoconjunctivitis.

Variable, n (%)	Palpebral (n = 56)	Limbal (n = 6)	Mixed (n = 16)	Total
Keratoconus	7 (9.0)	1 (1.3)	-	8 (10.3)
Subclinical keratoconus	5 (6.4)	1 (1.3)	3 (3.8)	9 (11.5)

Although male VKC subjects outnumbered females by 2:1, the prevalence of keratoconus among females (five eyes, 62.5%) was slightly higher than among males (three eyes, 37.5%). However, in contrast, male VKC subjects (eight eyes, 88.9%) with subclinical keratoconus greatly outnumbered females (one eye, 11.1%) by 8:1.

## Discussion

Due to VKC being a chronic conjunctival disease with corneal involvement, corneal topography has emerged as an essential tool for screening and detecting early changes and identifying subjects at risk prior to more progressive, irreversible changes. The ease of use and accuracy of modern corneal topographers, including Schiempflug topographers, allow quick and comfortable readings to be taken with minimal patient discomfort, thus facilitating the management of these VKC patients.

Males with VKC (53) outnumbered females (25) by 2:1 in our study. Furthermore, most VKC subjects were aged 10 years or less (33.3%), followed by 11-15 years (26.9%). The number of VKC subjects steadily reduced as the subjects’ age increased. This was consistent with the clinical course of VKC itself, which usually resolves by puberty [2]. A majority of subjects were Malay which was demographically consistent with the ethnicity distribution in Malaysia.

The palpebral type of VKC was the most common in our study (71.8%), followed by the mixed (20.5%) and limbal types. A similar distribution was reported in an epidemiological study conducted in Malaysia where the palpebral type was predominant, followed by mixed and none had limbal type alone [20]. These findings contrast previous studies, which showed the limbal and mixed types to be more common in Asia [1,4,13,14,16]. However, the South Asians in those studies were more heavily pigmented than the Southeast Asians in this study. This is pertinent because the density of mast cells and melanocytes were found to contribute to limbal VKC in dark-skinned individuals [4].

Greater than half of the study subjects had a VKC duration of five years or less (57.7%), with 82.1% being 10 years or less. This is unsurprising as VKC is a self-limiting condition that usually resolves by puberty, with only a few progressing to the chronic adult form of atopic keratoconjunctivitis [2].

Most subjects presented between the ages of six and 10 years (44.9%). This was consistent with the epidemiology of VKC itself, which predominantly affects males younger than 10 years [2,3].

VKC subjects showed significantly higher steep K2 (p = 0.001), Km (p = 0.021), and astigmatism (p < 0.001) compared to normal subjects. This concurs with other studies using both Placido-based and slit-scanning topographers [13,15,17]. These changes can be explained by the excessive eye-rubbing in VKC, which contributes to corneal remodeling, leading to increased curvature and progressive thinning, thus reiterating the clinical need to stop the eye-rubbing and optimize treatment to prevent permanent corneal remodeling [7]. Although the minimal pachymetry was found to be thinner in VKC compared to normal subjects, similar to studies by Gautam et al. and Baretto et al., this difference was not statistically significant [13,17]. There was no difference in flat K1, FDE, and BDE between both groups in contrast to the study using a slit-scanning topographer [17] (Table 8).

**Table 8:** Results of corneal topography in VKC CCT, central corneal thickness; α, directly proportional to; VKC, vernal keratoconjunctivitis.

Study	Country	Type of corneal topographer	Corneal topography
Our study	Malaysia	Schiempflug	Higher K_2_, K_m_,and astigmatism. Longer duration α thinner cornea. Older age of presentation α higher corneal curvature.
Gautam et al. [13]	Nepal	Placido	Reduced CCT, higher corneal curvature and astigmatism.
Totan et al. [14]	Turkey	Placido	-
Lapid-Gortzak et al. [15]	Israel	Placido	Higher K_max._ No correlation between type, severity, and duration.
Gupta et al. [16]	India	Placido	Longer duration α higher corneal curvature.
Baretto et al. [17]	Brazil	Slit-scanning	Higher anterior and posterior elevation peaks, thinner cornea, and increased corneal curvature.

Subgroup analysis of the corneal topographical changes based on the type of VKC showed no significant results despite the limbal type consistently demonstrating steeper corneal curvatures, thinner pachymetry, and higher front and back elevation peaks. This could be attributed to the small sample size available for the limbal type (six eyes) compared to the palpebral (56 eyes) and mixed (16 eyes) types. The only other study with a similar analysis showed the same conclusion due to the limitation of sample size after stratification [15]. Nevertheless, VKC classification remains essential to identify the limbal type as it tends to have more severe corneal topographical changes. Hence, more aggressive treatment should be initiated in this subtype.

A longer duration of VKC was directly proportional to thinner corneas (p = 0.039), whereby subjects with VKC of more than 15 years had a minimal pachymetry of 501 μm (IQR 68) when compared with those with VKC for only five years or less who had a minimal pachymetry of 546 μm (IQR 45). This is in contrast to a study in Israel which showed no significant difference regardless of the duration of VKC despite having a similar sample size to ours [15]. Mechanical trauma and the release of matrix metalloproteinases are thought to contribute to the progressive corneal thinning found in VKC subjects, and a longer duration of VKC correlates to more severe forms of the disease [7,8]. However, there was no significant difference between the duration of VKC and corneal curvature, FDE, BDE, and astigmatism.

VKC subjects with older age of presentation were associated with steeper K1 (p = 0.040), K2 (p = 0.028), and Km (p = 0.014) and thinner minimal pachymetry (p = 0.006). These may be attributed to the longer duration of untreated VKC leading to more corneal damage. Since there were differences in the corneal topography between the different subtypes of VKC, future studies should involve patients of only one subtype for a more reliable comparison.

87.5% (seven eyes) of keratoconus patients belonged to the palpebral type of VKC, followed by the limbal type with 12.5% (one eye). This finding mirrored a study by Chingu et al. in Turkey, where 60% of keratoconus subjects with VKC had the palpebral type, followed by 28.5% and 11.4% with mixed and limbal types, respectively [9] (Table 9). The rate of subclinical keratoconus was slightly higher (11.5%) than that of keratoconus (10.3%), which was comparable to other studies [13-15]. However, this was in contrast to the study using slit-scanning topography, which showed a higher prevalence of keratoconus (20.0%) compared to subclinical keratoconus (14.0%) [17] (Table 9).

**Table 9:** Results of the prevalence of keratoconus in VKC VKC, vernal keratoconjunctivitis.

Study	Country	Prevalence of keratoconus (%)	Prevalence of subclinical keratoconus (%)
Our study	Malaysia	10.3	11.5
Gautam et al. [13]	Nepal	1.7	11.3
Totan et al. [14]	Turkey	8.5	26.8
Lapid-Gortzak et al. [15]	Israel	4.0	16.0
Gupta et al. [16]	India	8.0	-
Baretto et al. [17]	Brazil	20.0	14.0

The limitation of this study was that eyes with more severe forms of VKC were excluded due to very poor ocular surface disease, the presence of corneal scars, or previous surgical intervention, as they would adversely affect the corneal topography.

## Conclusions

Longer duration and older age of VKC presentation are associated with more significant corneal topographical changes, thus leading to a higher risk of keratoconus. The prevalence of keratoconus and subclinical keratoconus among VKC subjects is similar at 10%. Corneal topography of at-risk VKC subjects offers a cost-effective modality in reducing future morbidity with education and tailored treatment.
